# CurraNZ blackcurrant improves cycling performance and recovery in trained endurance athletes

**DOI:** 10.1186/1550-2783-11-S1-P14

**Published:** 2014-12-01

**Authors:** Mark ET Willems, Stephen D Myers, Sam D Blacker, Matthew D Cook

**Affiliations:** 1 University of Chichester, Chichester, United Kingdom

## Background

Peripheral blood flow is increased by blackcurrant intake in humans [1], potentially by anthocyanin-induced vasorelaxation and vasodilation [2], which may affect substrate delivery, exercise performance and recovery. We examined the effect of 1-week CurraNZ blackcurrant on substrate oxidation during steady state cycling, 16.1 km (10 mile) time-trial performance and lactate clearance following exercise in trained endurance athletes.

## Methods

Nine male endurance athletes (club level cyclists and triathletes with >3 yrs experience; age: 35±14 years, height: 179±3 cm, body mass: 76±9 kg, BMI: 24±2, VO2max: 54±6 mL kg-1 min-1, maximum power: 366±42 W, mean±SD) volunteered to visit the laboratory for 4 sessions. Cycling tests for lactate responses (4 min stages with 2 min recovery, start power 50 W with 30 W increments) and maximum oxygen uptake (start power 50 W for 4 min with 30 W min-1 increments) at self-selected pedal cadence (SRM ergometer, SRM International, Germany) were performed to establish power values at 45%, 55%, and 65% of VO2max. Experimental design was double-blind and randomized with a wash-out period of 2 weeks. Familiarized participants were tested following 7 days of blackcurrant extract (CurraNZ, 300mg/day) (Health Currancy Ltd, UK) or placebo (P) capsule intake. Indirect calorimetry (Douglas bag technique) was used at low (~45%) and moderate intensity (~55% and ~65%) steady-state cycling (10 min stages) with lactate sampling. Subsequently, a 16.1 km time-trial was performed with lactate sampling during recovery for 20 min. Paired t-tests were used for analysis with significance accepted at p<.05. Consent to publish the results was obtained from all participants.

## Results

There were no differences between CurraNZ and placebo at ~45%, ~55% and ~65% VO2max for fat oxidation, carbohydrate oxidation, lactate, heart rate, minute ventilation and cycling economy (P>.05). CurraNZ improved 16.1 km time-trial performance substantially by 3.6% (P: 1784±121, CurraNZ: 1718±108 sec, p=.03, 7 out of 9 participants improved, range -2.2-8.6%). Lactate was higher with CurraNZ immediately following the time-trial (P: 5.4±1.6, CurraNZ: 6.5±1.8 mmol L-1, p=.03, all participants). Lactate decreases were higher with CurraNZ after 20 min of passive recovery following the time-trial (P: 3.2±0.8, CurraNZ: 3.9±1.2 mmol L-1, p=.03, 8 out of 9 participants).

## Conclusions

Intake of CurraNZ blackcurrant is associated with 1) normal metabolic and physiological responses at low and moderate intensity cycling, 2) improved 16.1 km (10 mile) time-trial cycling performance, 3) potentially a higher lactate tolerance during time-trial performance, and 4) increased lactate clearance after exercise indicating improved recovery. It is concluded that CurraNZ blackcurrant intake has favourable implications in endurance athletes for aerobic exercise performance, lactate tolerance, and recovery.
